# Plum Fruit Development Occurs via Gibberellin–Sensitive and –Insensitive DELLA Repressors

**DOI:** 10.1371/journal.pone.0169440

**Published:** 2017-01-11

**Authors:** Islam El-Sharkawy, Sherif Sherif, Mahboob Abdulla, Subramanian Jayasankar

**Affiliations:** 1 Florida A&M University, Center of Viticulture and Small Fruit Research, Tallahassee, Florida, United States of America; 2 Faculty of Agriculture, Damanhour University, Damanhour, Egypt; 3 University of Guelph, Department of Plant Agriculture, Ontario, Canada; 4 Department of Chemistry, School of Science and Technology, Nazarbayev University, Astana, Kazakhstan; Wuhan Botanical Garden, CHINA

## Abstract

Fruit growth depends on highly coordinated hormonal activities. The phytohormone gibberellin (GA) promotes growth by triggering degradation of the growth-repressing DELLA proteins; however, the extent to which such proteins contribute to GA-mediated fruit development remains to be clarified. Three new plum genes encoding DELLA proteins, PslGAI, PslRGL and PslRGA were isolated and functionally characterized. Analysis of expression profile during fruit development suggested that *PslDELLA* are transcriptionally regulated during flower and fruit ontogeny with potential positive regulation by GA and ethylene, depending on organ and developmental stage. PslGAI and PslRGL deduced proteins contain all domains present in typical DELLA proteins. However, PslRGA exhibited a degenerated DELLA domain and subsequently lacks in GID1–DELLA interaction property. *PslDELLA*–overexpression in WT *Arabidopsis* caused dramatic disruption in overall growth including root length, stem elongation, plant architecture, flower structure, fertility, and considerable retardation in development due to dramatic distortion in GA-metabolic pathway. GA treatment enhanced PslGAI/PslRGL interaction with PslGID1 receptors, causing protein destabilization and relief of growth-restraining effect. By contrast, PslRGA protein was not degraded by GA due to its inability to interact with PslGID1. Relative to other *PslDELLA*–mutants, *PslRGA*–plants displayed stronger constitutive repressive growth that was irreversible by GA application. The present results describe additional complexities in GA-signalling during plum fruit development, which may be particularly important to optimize successful reproductive growth.

## Introduction

Fruit development is a multiphase process that requires a tight coordination of molecular, biochemical and structural elements. The series of modifications that control the transition of fruit growth through consequent developmental stages involve many distinctive metabolic pathways. In recent years, many molecular and genetic mechanisms underlying the action of phytohormones in fruit development have been identified, uncovering the complexity of this regulatory network [1–4]. Collectively, hormone application, endogenous hormone quantification and genetic studies support the hypothesis that fruit development is largely coordinated by hormonal interplay. Gibberellin (GA) is an essential hormone involved in diverse biological processes, leading to correct plant growth and development [5–8]. In tree fruit species, the proper establishment of reproductive growth is dependent on coordinated levels of GA at the appropriate developmental stages [9–11]. Application of GA resulted in visible improvement of fruit quality traits in terms of size, weight and many other characteristics [12–13]. Conversely, mutant fruits with inadequate quantities of GA exhibited a series of distortions in floral development and general reproductive growth events [1–2, 9, 14]. Although the potential impact of GA in coordinating fruit development processes has already been acknowledged [15–18], the mechanism by which these effects are achieved is still largely unknown. This may be due to the diversity of cross-talk between GA and other hormones, which are often species/organ/developmental stage-dependent [19]. Several lines of evidence point out the essential role of GA in coordinating reproductive growth. In flowering plants, GA specifies the site of floral primordium initiation, and acts with homeotic genes to ensure proper floral organogenesis and patterning [20–23]. Molecular and genetic studies highlighted the pivotal contribution of GA during fruit-set, the term given to the onset of rapid cell division necessary for early embryo development and fruiting structure enlargement [1–2]. The transition of ovary into fruit, initiated upon successful fertilization, activates GA pathway in the ovules that acts with other hormones, particularly auxin and cytokinin, in triggering fruit-set program, thereby stimulating fruit growth [2, 4, 24–27]. Previous studies have shown that the endogenous GA content readily increased along with the progression in fruit maturity and ripening [13, 15, 28]. These findings coupled with the stimulatory effect of exogenous GA in the fruit growth of several species suggested that GA is needed in mature fruiting tissues to allow fruit enlargement with potential involvement in ripening [1, 12–13, 29–31]. On the other hand, the scarcity of bioactive GA during plum fruit growth caused serious developmental disorders, including growth retardation, disturbed flower patterning and limited fruit characteristics [9].

Insight into mechanisms of GA-regulated plant development has been manifested from research into GA-biosynthesis, -metabolism and -signalling pathways [19, 32]. The major metabolic processes regulating GA-biosynthesis and -deactivation have been identified [33]. By contrast, the discovery of GA-receptors and downstream signalling components has been recently elucidated [34–36]. The central of GA-signalling are the DELLA proteins that are part of the wider GRAS family of regulatory proteins [37]. According to the relief of restraint model, DELLA proteins operate as growth-repressors and GA-mediated DELLA degradation is a critical step to overcome this restraint [38]. In agreement with their function as growth-repressors, lacking one or more of DELLA proteins within the plant elicited constitutive activation of GA-signalling pathway independent to the hormone presence in which the mutant plants exhibited GA-overdosed phenotype, including slender vegetative growth and parthenocarpic fruit development [39–44]. At low GA levels, DELLA proteins impair the activity of basic helix-loop-helix transcription factors by interacting with their DNA binding domain [45–46]. The binding of GA to its receptor GIBBERELLIN-INSENSITIVE DWARF1 (GID1) results in a conformational change that promotes interaction of GID1 with the DELLA domain of DELLA proteins [47–50]. The GA–GID1–DELLA complex is subsequently recognized by the SCF^SLY1/GID2^ E3 ubiquitin-ligase complex, which mediates ubiquitination of DELLA proteins. This ubiquitin mark destines the DELLA proteins for degradation via the 26S proteasome, thereby allowing growth by releasing their inhibitory interaction with GA-dependent gene partners [45–46, 51–55].

In plants, it is important to maintain optimal levels of hormone signalling to ensure normal growth. Disruption of this signalling pathway can dramatically impact plant development. Nevertheless, the severity of these phenotypic changes can vary within and among species [56–61]. For instance, the altered phenotype in some GA-deficient mutants can be easily reversed to normal by applying external GA, while others show unresponsive effect to GA treatment [62].

In the present study, three novel genes encoding DELLA proteins were isolated from Japanese plum cultivar Early Golden (*Prunus salicina* L.). To understand the potential involvement of various *PslDELLA* in fruit growth, their expression profile was assessed throughout fruit development. We next investigated PslDELLA function to provide evidence that the identified proteins are responsible for regulating the GA-responsiveness during fruit growth. Sequence analysis indicated that PslGAI and PslRGL deduced proteins contain all domains present in typical DELLA proteins; however, PslRGA lack the intact DELLA domain necessary for the GA-dependent interaction with GA-receptors, GID1. Despite this fact, PslRGA primary structure showed high similarity to the *C*-terminal portions of DELLA proteins, and phylogenetic and modelling structure classified it as a member of DELLA group. Analysis of yeast two-hybrid (Y2H) and bimolecular fluorescence complementation (BiFC) assays indicated that PslGAI and PslRGL proteins are active repressor components that effectively interact with PslGID1 receptors in a GA-dependent manner. However, PslRGA was not able to form complex with PslGID1 proteins under different circumstances of GA-types or concentrations. Phenotypical analysis of transgenic *Arabidopsis* plants overexpressing each of *PslDELLA* confirmed the function of the three proteins as growth-repressors. Although *PslGAI*–and *PslRGL*–mutant plants were able to recover the normal growth by GA application, *PslRGA*–plants exhibited constitutive inhibition of GA-signalling, overcoming the destabilization effect of GA. Finally, we provided several lines of evidence that *PslRGA* encode a strong stable DELLA protein independent of GA action and this was mainly due to critical substitutions occurring within the essential DELLA domain.

## Materials and Methods

### Plum tissues and treatments

Flowers and fruits from sequential developmental stages were harvested from Japanese plum cultivar Early Golden (EG) as described previously [64]. Since the seed is inseparable in S1 and S2 growth phases, the whole fruit tissue was used for RNA extraction, while in S3 and S4 stages the pulp tissue was carefully separated from the seed for RNA analysis. To evaluate the potential ethylene-dependent regulation of *PslDELLA* during plum fruit ripening, mature EG fruit (76 DAB) were harvested before autocatalytic ethylene production had risen, surface sterilized, and subjected to various treatments. These included propylene (1000 μl l^–1^), the ethylene-inhibitor 1-MCP (1 μl l^–1^) and water-dipped fruit were used as control. Fruit were sampled at different stages of ethylene production (non-climacteric, pre-climacteric, climacteric and post-climacteric), by assessing ethylene evolution. In 1-MCP treatment samples were collected at similar age to that of control fruit. In all cases, mixed tissues of at least twelve fruit (distributed into 3 biological replicates) at the same age or displaying a similar ethylene production were used for mRNA extraction and analysis. All samples were frozen in liquid nitrogen immediately after collection and stored at −80°C.

### Isolation and in silico analysis of PslDELLA sequences

Based on the sequence similarity among various *DELLA* cDNAs, a pair of degenerate primers (S1 Table) was designed in the conserved regions to amplify the plum orthologs from EG cDNA under stringent primer hybridization conditions. Fragments from several independent PCR reactions were cloned, sequenced and compared with database sequences using the BLAST program [65]. Extension of the partial cDNA clones were carried out using the 5’- and 3’- RACE kit (Invitrogen, Burlington, ON, Canada). Full-length amplification of cDNA sequences designated *PslGAI*, *PslRGL* and *PslRGA* was carried out using Platinum Taq DNA Polymerase High Fidelity, following the instructions provided by the manufacturer (Invitrogen). The names of the individual plum *DELLA* introduced here are not intended to imply functional homology to specific *Arabidopsis* DELLA protein. Since there is two different alleles of *PslRGL* and *PslRGA* (*a* & *b*), unless mentioned otherwise *PslRGL* and *PslRGA* will be always referred to *PslRGLa* and *PslRGAa*, respectively. The group of the three genes and proteins PslGAI, PslRGLa and PslRGAa are referred to as *PslDELLA* and PslDELLA, respectively. Alignment of predicted proteins was performed using ClustalX and the neighbor-joining tree was generated with MEGA5 [66]. Full-length genomic sequences were isolated using the AccuPrime *Pfx* (Invitrogen). To determine the function of *PslRGA* sequence, mutated version of *PslRGL* and *PslRGA* designated *PslRGL*_.*MU*_ and *PslRGA*_.*MU*_, respectively; were generated using the QuikChange site-directed mutagenesis kit (Stratagene, San Diego, CA, USA). Changes were generated within the N-terminal DELLA and TVHYNP motifs of *PslRGL* sequence to mimic *PslRGA* and conversely in *PslRGA* to simulate that of *PslRGL* sequence.

### DNA, RNA extractions and qPCR assays

Genomic DNA was extracted from young plum leaves according to the DNeasy Plant Maxi Kit (Qiagen, Mississauga, ON, Canada). Total RNA extraction, DNase treatment, cDNA synthesis, and qPCR reactions were performed as described previously [9]. Gene-specific primers were designed using Primer Express (v3.0, Applied Biosystems, Carlsbad, CA, USA) (S1 Table). Three independent biological replicates for each reaction were run on an ABI PRISM 7900HT Sequence Detection System (Applied Biosystems) and each experiment was repeated three times. Transcript abundance was quantified using standard curves for both target and reference genes [*PslAct* (EF585293), *AtAct* (NM_121018)], which were generated from serial dilutions of PCR products from corresponding cDNAs. The data were present as an average ±SD.

### Observation of GFP fluorescence

Full-length coding sequences of *PslGAI*, *PslRGL*, *PslRGA*, and the mutated *PslRGL*_.*MU*_ and *PslRGA*_.*MU*_ versions were fused in frame with the GFP into the pGreenII vector using the *Bam*HI site and expressed under the control of the 35S promoter. For protoplasts assay, the different constructs were transfected into protoplasts from suspension cultured tobacco BY-2 cells exposed to different treatments that alter GA-response, including 100 μM GA_3_ and/or 10 μM of GA-biosynthesis inhibitor paclobutrazol (PAC). Non-treated protoplasts were used as a mock control. For *Arabidopsis* assay, transgenic seeds independently expressing the different PslDELLA−GFP chimeric proteins (excluding PslRGL_.MU_) were germinated in standard MS growth medium. After 5 days, the roots were observed initially for GFP fluorescence and then exposed to respective treatments as described above for 60, 120 and 240 minutes. After each time point, root samples were mounted on microscope slides and analyzed for GFP fluorescence using confocal microscopy as described previously [67]. All assays were repeated three times.

### Protein structure prediction

The three-dimensional (3-D) crystal structures of *Arabidopsis* GA_3_–GID1–DELLA complex (PDB ID: 2ZSH) was used as a template to obtain homology models of PslDELLA proteins by the MODELLER package. The resulting structures were optimized using the generalized born model for solvent of Amber12 software package. The binding energy of the respective PslDELLA proteins to individual GA_3_–PslGID1 complex was then calculated through obtaining the electrostatic components of the thermodynamic cycle corresponding to a protein–protein binding event [68]. The electrostatic energies were calculated using the numerical Poisson–Boltzmann solver algorithm APBS software version 1.3 [69].

### Bimolecular fluorescence complementation (BiFC) assay

For constructs used in the BiFC experiment, the *N*-terminal (pSAT1-N) and *C*-terminal (pSAT1-C) EYFP vectors were used. The full-length of all *PslDELLA*, including the mutated *PslRGL*_.*MU*_ and *PslRGA*_.*MU*_ versions were fused into the *Sac*II-*Bam*HI site of the pSAT1-C vector. Consequently, plum GA-receptors *PslGID1b* and *1c* were inserted into the *Bgl*II-*Bam*HI of the pSAT1-N vector. The different combinations of constructs encoding NY and CY at similar concentrations were mixed and then co-transfected into protoplasts obtained from suspension-cultured tobacco BY-2 cells in the presence or absence of 100 μM GA_3_, as described previously [13]. All assays were repeated at least three times and visualized using confocal microscopy.

### Yeast two-hybrid (Y2H) assays

Y2H assays were performed with the Matchmaker Gold Yeast two-hybrid System (Clontech, Palo Alto, CA, USA). *PslDELLA* full-length ORFs and the mutated *PslRGL*_.*MU*_ and *PslRGA*_.*MU*_ versions were inserted into the *Nde*I-*Bam*HI site of the pGADT7 prey vector (GAL4 activation-domain; AD). *PslGID1b* and *1c* cDNAs were fused into the *Bam*HI-*Pst*I and *Nde*I-*BamH*I sites of the pGBKT7 bait vector (GAL4 binding-domain; DBD), respectively. Prey and bait vectors (100 ng) were then introduced into Y2HGold and Y187 yeast strains, respectively; using Yeastmaker yeast transformation system 2. Interactions between the proteins were assayed by the mating method, according to the manufacturer’s instructions, in 96-well plates containing medium with or without 100 μM GA (GA_1_, GA_3_ or GA_4_), as described previously [13]. All assays were repeated at least three independent times.

### Plasmid construction and plant transformation

Full-length *PslDELLA* and *PslRGA*_.*MU*_ (excluding the stop codon) were fused with the GFP reporter gene in the binary vector pGreen0029 [70]. The resulting vectors were transformed into *A*. *tumefaciens* and employed for *Arabidopsis* transformation, as described previously [9]. All genes under the control of the *35S* promoter were introduced into wild-type (WT) *Arabidopsis* background Col-0. Non-transformed WT plants as well as plants transformed with empty vectors were used as controls. Different generations of transgenic plants were selected under kanamycin resistance circumstances. T3 homozygous independent lines from each transformation were grown under standard long day conditions (16:8 h light/300 μmol m^−2^ s^−1^; 23:18°C and 65% relative humidity) with or without GA_3_ treatment; 24 plants/transformation/treatment. All plant materials were frozen and stored at −80°C until use.

## Results and Discussion

### Isolation and structural characterization of *PslDELLA* cDNAs

**Fig 1:**
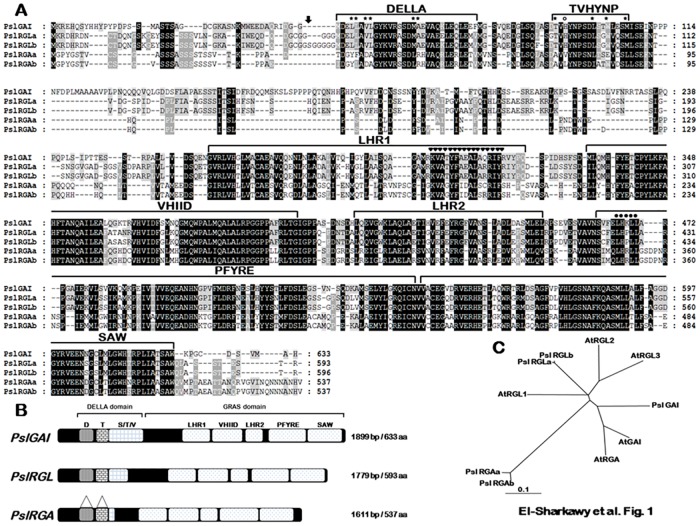
(**A**) Amino acid sequence alignment of plum *DELLAs PslGAI* (KU845589), *PslRGLa/b* (KU845592/KU845593), and *PslRGAa/b* (KU845590/KU845591) using ClustalX program. Conserved residues are shaded in black. Dark- and clear-grey shadings indicate similar residues in four and three out of five of the sequences, respectively. Conserved motifs are shown above the alignment columns. A putative nuclear localization signal (NLS) is indicated by black triangles. Conserved LXXLL motif is indicated by black circles. Asterix and open circle within DELLA/TVHYNP motifs highlight the substituted amino acid residues in *PslRGAa*/*b* that are essential for interaction with the GID1-like proteins and complex stabilization, respectively. The arrow indicates the site of the three amino acid residues insertion (Ser-Gly-Gly) in *PslRGLb* (**B**) Schematic representation of *PslGAI*, *PslRGL* and *PslRGA* proteins domain organization. The triangles in *PslRGA* are to highlight the location of distinct DELLA motifs. The GA-responsive DELLA domain [DELLA (D) and TVHYNP (T) motifs], the poly STV (S/T/V) motif, and the functional GRAS domain [LHR1, VHIID, LHR2, PFYRE and SAW motifs] are indicated. Number of base pairs (bp) and amino acids (aa) refer to full-length nucleotides and amino acid residues of the predicted sequences. (**C**) Phylogenetic relationships between PslDELLAs and *Arabidopsis* orthologous (AtGAI, AtRGA, AtRGL1, AtRGL2 and AtRGL3). The tree was constructed using MEGA5 software. The scale bar represents a number of amino acid substitutions per site, in which 1 cm is equal to 0.1 amino acid substitutions per site.

To investigate the molecular basis of GA action in fruit development, three novel sequences closely related to the growth-repressing DELLA proteins, a subset of the plant-specific GRAS (GAI, RGA and SCARECROW) family of transcriptional regulators were isolated from Early Golden (EG) plum cultivar. PslGAI, PslRGL, and PslRGA predicted to encode proteins of 633, 593, and 537 amino acid residues with calculated molecular weights of 69.9, 64.5, and 59 kDa, respectively. The relationships between the predicted plum and *Arabidopsis* amino acid sequences, as indicated by percentage similarity over the whole sequence, are presented in S2 Table. The various *PslDELLA* showed considerable sequence deviation (50–70% similarity), mainly due to the divergence of *N*-terminal portions outside the DELLA domain. Nevertheless, several signature structural elements commonly associated with the DELLA subfamily were detected (Fig 1A and 1B). The deduced amino acid sequences of *PslGAI* and *PslRGL* comprise the two domains essential for the protein function, including the typical *N*-terminal DELLA domain (DELLA and TVHYNP motifs) and the highly conserved *C*-terminal GRAS domain. Both domains are necessary for the GA-dependent interaction with GA-receptors GID1 and involved in the repression function of the protein [71–72]. Although *PslRGA* sequence displayed structurally high similarity with other DELLA proteins, critical divergences in the key amino acid residues important for GID1–DELLA interactions were detected [50], resulting in partially conserved DELLA domain (Fig 1A). For instance, in the DELLA motif, comprising De**LL**a**ΦL**xYxV sequence; the three Leu residues are substituted by the distinct amino acids Tyr, Phe and Ala in PslRGA. While the nonpolar residue represented by Φ (Val_52_ and Val_53_ in PslGAI and PslRGL, respectively) is replaced by Asp_37_ in PslRGA. Further, in the following LExLE motif with the consensus sequence **MA**xVAxxLExLExΦ; the first amino acid Met is substituted by Leu and the essential nonpolar Ala residue is changed into Arg. Finally, in the TVHYNP motif (**T**V**h**ynPxxLxx**W**xxx**M**), the amino acid residues Thr, Try, and Met are substituted by Ala, Glu, and Leu, respectively. While the amino acid His that is not important for GID1 interaction, but contributes to protein stabilization [73] is changed into Val. All these critical alterations in amino acid residues essential for the direct GID1 interaction surface suggested that PslRGA may function differentially by having a distinct interaction mode with GID1-like proteins in comparison with typical proteins holding conserved DELLA domain.

In an attempt to unravel the genomic and allelotype structure of the different *PslDELLA* genes, the full-length genomic sequences of the three genes were isolated and sequenced from EG *g*DNA. Consistent with *DELLA* gene subfamily, all plum genes exhibited a single open reading frame without any intron interruption. EG is homozygous for *PslGAI*; however, two different alleles were identified for *PslRGL* (a & b) and *PslRGA* (a & b) with non-synonymous alterations in nucleotide composition, leading to several changes in the predicted proteins (Fig 1A). The two *PslRGL* and *PslRGA* alleles share 98% and 94% amino acid sequence identity, respectively; reflecting the presumed allopolyploid origins. One of the most significant differences between the two *PslRGL* alleles is the detection of a microsatellite region with imperfect nucleotide recreates due to the insertion of three amino acid residues Ser-Gly-Gly within the *N*-terminal region of *PslRGLb* at position 44. On the other side, all the critical structural changes within the *N*-terminal DELLA domain of *PslRGAa* were detectable in *PslRGAb* allele. Sequence data mining in *Prunus* species genome (e.g. *P*. *persica* and *P*. *mume*), the closest genomes to plum (*P*. *salicina*), identified the three *PslDELLA* as the only putative *DELLA*-like genes within the genome.

Phylogenetic analysis of *PslDELLA* with closely related genes from *Arabidopsis* indicated that *PslGAI* can be grouped into the clade of *AtGAI* and *AtRGA*, whereas *PslRGLa* and *b* are clustered with *AtRGL*-related proteins (Fig 1C). Interestingly, *PslRGAa* and *b* form a unique distant clade without any representative from *Arabidopsis* orthologous, indicating that this clade may represent a new branch in DELLA protein evolution. Nonetheless, sequence data mining identified members closely related to *PslRGA* clade in many other plant species (S1 Fig).

To gain a broader insight into PslDELLA function, we investigated the localization compartment of their proteins. Fluorescence microscopy revealed that, as expected, the full-length PslDELLA−GFP fusions were localized exclusively in the nucleus (S2 Fig), which is consistent with their primary function as transcription regulators [74–75]. Together, these primary comparative analyses suggested that PslGAI and PslRGL might be involved in GA-signalling in a manner similar to that of other characteristic DELLA proteins via interaction with the GA-receptor GID1-like proteins [48]. However, the distinct PslRGA protein might exhibit particular function due to the disrupted DELLA domain. This hypothesis was tested using a number of biochemical and biological approaches, as described below.

### Molecular modelling of PslDELLA proteins

To determine whether the putative plum proteins exhibited similar function to those of *Arabidopsis* DELLA, three-dimensional modelling of PslDELLA proteins was generated and compared with AtGAI as a template. Analysis of the predicted structures indicated that all PslDELLA proteins are highly similar to AtGAI protein (S3 Fig). The two typical DELLA and GRAS domains as well as the links between the domains constituted the differences among the four proteins. Although PslGAI and PslRGL exhibited few amino acid alterations within the *N*-terminal DELLA domain, these changes are not in the contact residues with GID1-like proteins, but rather in the amino acids that contribute to the conformation of the protein in this area. In the meantime, the *3-D* structures did not establish a clear difference between the divergent PslRGA protein and the other DELLA proteins holding complete domain. This is probably due to the conserved long *C*-terminal GRAS domain within the structure. Consequently, the binding energies of the three PslDELLA to the previously characterized plum GA-receptors PslGID1b and 1c [13] in the presence of GA_3_ molecule were calculated to determine the PslDELLA’s interaction capacities. Data analysis revealed that the three PslDELLA proteins displayed differential binding energy features to the different PslGID1-like proteins. The binding energies of PslGAI and PslRGL to PslGID1s were found to be within the range of −3 and −17 kcal mol^−1^, respectively. However, PslRGA did not show any obvious binding ability for either of PslGID1 proteins with binding energy estimated at ~23 kcal mol^−1^.

### Properties of PslGID1–PslDELLA interaction

**Fig 2:**
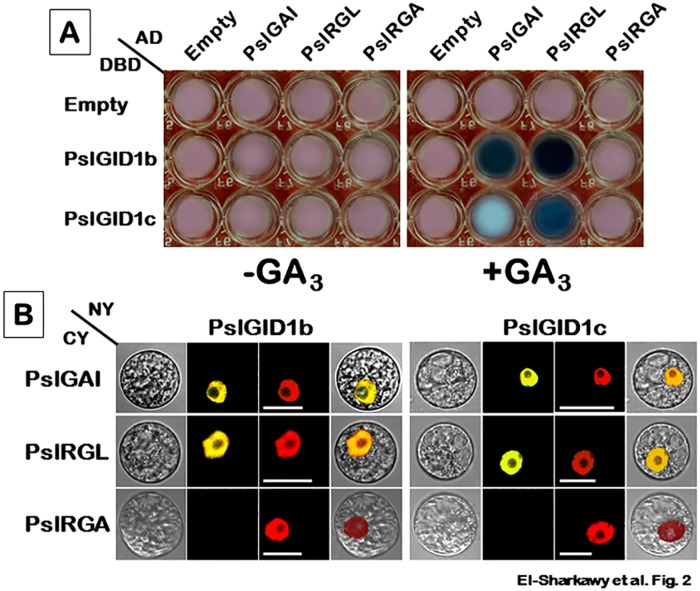
Interaction capacity between PslDELLA (PslGAI, PslRGL and PslRGA) and GA-receptors (PslGID1b and PslGID1c) using Y2H and BiFC approaches. Y2H assays (**A**) were performed using PslDELLA as prey in Y187 yeast strain and PslGID1 as bait in Y2HGold yeast strain. The mated yeast was grown in 96-well plates containing DDO/X/A medium in the presence or absence of 100 μM GA_3_. For *in planta* BiFC assay (**B**), PslDELLA sequences were fused with the *C*-terminus (CY) of YFP; PslGID1 were fused with the *N*-terminus (NY) of YFP. Different combinations of NY and CY constructs were transiently co-expressed in GA-treated tobacco protoplasts. *NLS*-mCherry was included in each transfection to highlight the location of the nucleus. YFP fluorescence is yellow; the merged image is a digital merge of bright field and fluorescent images to illustrate the interaction location; bars = 10 μm. All experiments were repeated at least three times.

Considerable progress has been made in elucidating the molecular basis of GA action [8]. Perception of bioactive GA by its GID1 receptors promotes the direct interactions between GID1 and DELLA domain of GA-repressors DELLA [76–77]. To determine whether PslDELLA possess a comparable function as those of *Arabidopsis*, the interactions between PslGID1 and PslDELLA were assessed in yeast system in the presence or absence of GA_3_ (Fig 2A). The analysis revealed a clear divergence in terms of the interaction capacity and preference. Previous studies have shown that interactions between the *Arabidopsis* GID1 and DELLA are enhanced in yeast cells in the presence of bioactive GA [47]. Similarly, the binding results confirmed the essential GA-induced assembly of stable GA–PslGID1–PslDELLA complex in yeast. PslGAI and PslRGL were effectively able to interact with both PslGID1s; however, they showed differential binding efficacy to a specific PslGID1 protein. Their capacity to form complex with PslGID1b was much stronger than PslGID1c. Conversely, PslRGA protein did not bind to any of PslGID1s, even in the presence of GA. It has been reported that the two PslGID1s perfectly interacted with *Arabidopsis* GAI and RGL1 proteins in yeast system [13]. Nevertheless, this may be different in the case of PslGID1 and PslDELLA, where sequence and conformational differences may confer some levels of specificity in PslGID1–PslDELLA pairing.

To provide additional evidence, we attempted to visualize the direct GA-triggered interactions between PslGID1 and PslDELLA using BiFC approach. Tobacco protoplasts supplemented with 0 and 100 μM GA_3_ were co-transfected with the various combinations of NY–PslGID1 and CY–PslDELLA constructs (Fig 2B). The YFP signal caused by interaction between PslGID1 and PslDELLA was only detected in protoplasts pre-treated with GA. Although, the untreated cells should contain endogenous GA, no fluorescence signals were observed in cells grown in GA-free medium (data not shown). This is probably due to the scarcity of active GA content that is not sufficient to promote interaction between the fluorescence-labeled PslGID1 and PslDELLA. Consistent with yeast assays, both PslGAI and PslRGL proteins exhibited high activity to form complexes with both PslGID1s; however, PslRGA did not interact with any of the PslGID1 proteins. GA orchestrates a broad range of processes and many levels of regulation are known to be involved in determining GA-responses, including biosynthesis, metabolism and signalling [8, 78]. Our data and those of others [11, 79] showed that another level of regulation exists in terms of GA–GID1–DELLA binding capacity and preference.

### PslGID1–PslDELLA interaction is GA–type-dependent

**Fig 3:**
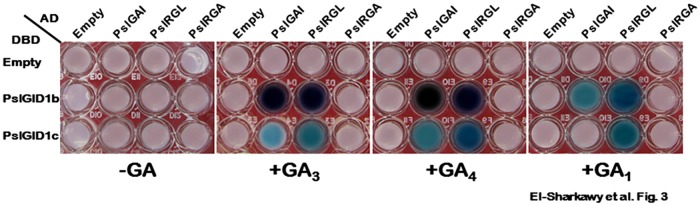
Stabilization of GA–PslGID1–PslDELLA complexes is dependent on the type of bioactive GA, mediating the reaction. Y2H interaction experiments of PslGAI, PslRGL and PslRGA with PslGID1b and PslGID1c on selective medium containing 100 μM GA_1_, GA_3_ and GA_4_. Selective medium without bioactive GA was used as control. Other details as in Fig 2.

It was reported that the interaction preference of GID1–DELLA proteins in yeast cells is dependent on the structural features of the bioactive GA used in the reaction [79]. Bioactive GAs, GA_1_, GA_3_ and GA_4_, share three common structural traits, including a hydroxyl group on C-3β, a carboxyl group on C-6, and a lactone between C-4 and C-10, in which the 3β-hydroxyl group can be exchanged for other functional groups at C-2 and/or C-3 positions [33]. However, GA_1_ and GA_3_ differ from GA_4_ by the presence of hydroxyl group at C-13 that can influence the activity of the GA structure [80]. Accordingly, it is possible to speculate that the reduced interaction activity of PslGAI and PslRGL with PslGID1c, and the lack of protein–protein interaction between PslRGA and both plum GA-receptors are due to using GA_3_ as a mediator of the reaction. To test this hypothesis, we examined the interaction property of different PslGID1–PslDELLA in the presence of GA_1_ and GA_4_; the most abundant bioactive GA involved in plum fruit development [13]. Interaction assays in GA-free and GA_3_-containing mediums were included as controls (Fig 3). The effect of GA_4_ on the PslGID1–PslGAI/PslRGL interactions was comparable to that of GA_3_ and the reduced interaction activity of the two PslDELLA proteins with PslGID1c remained detectable. However, GA_1_ showed generally lower activity in triggering the interaction between PslGAI/PslRGL and PslGID1s than GA_3_ or GA_4_, with no visible interaction between PslGAI and PslGID1c. Altogether, the results of Y2H experiments suggested that there is substrate preference among the PslGID1-like proteins, which further depends on the structure of bioactive GA. PslGAI and PslRGL are generally better substrates for PslGID1b than for PslGID1c. In the same time, bioactive GA_3_ and GA_4_ are better mediators for the interaction than GA_1_. One of the most outstanding questions in GA biology is how the hormone controls so many different aspects of plant growth and development. On the basis of Y2H results, it is possible that different GA-PslGID1–PslDELLA complexes have diverse biochemical properties that enable specialized functions.

Despite the type of GA tested, the interactions between PslRGA and PslGID1 proteins were undetectable, confirming the loss of interaction capability of that protein under different circumstances of bioactive GA in yeast cells. Conserved DELLA domain is essential for GA-GID1–DELLA interaction, since any deletion or point substitution results in loss-of-interaction ability despite the presence of GA [47, 49, 58, 75]. Recently, a grape DELLA protein, VvDELLA3, exhibiting high sequence similarity to PslRGA has been characterized [11]. Although, both VvDELLA3 and PslRGA share the disrupted DELLA domain, VvDELLA3 displayed selective interaction with VvGID1-like proteins. Further, the ability of VvDELLA3 protein degradation in response to GA treatment suggests its active function as a GA-sensitive repressor. Sequence comparison between PslRGA and VvDELLA3 highlighted more pivotal substitutions within the DELLA motifs that could potentially account for abolished interaction between PslRGA and PslGID1-like proteins (S4 Fig). By contrast, Fleck and Harberd [75] provide evidence that the distinct *Arabidopsis* DELLA proteins, GAI and RGL1, are GA-insensitive stable proteins, as they do not disappear from the nucleus in response to GA treatment and the plants overexpressing each of these two proteins exhibited dwarf, GA non-responsive phenotype.

### PslRGA substitutions abolish the GA-dependent complex formation with PslGID1s

**Fig 4:**
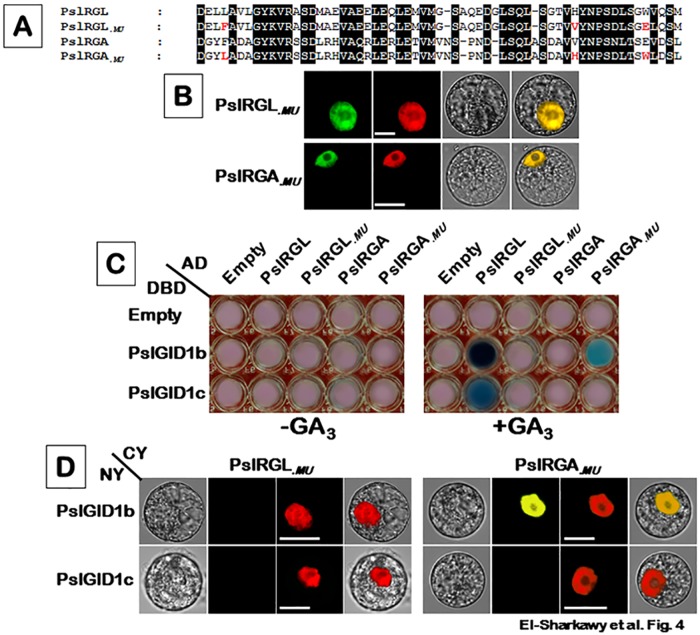
Substitutions in the DELLA domain of PslRGA protein prevent the GA-dependent interaction between PslRGA and PslGID1-like proteins. (**A**) Alignment of amino acid sequences of the GA-sensitive *PslRGL*, GA-insensitive *PslRGA* and their mutated versions *PslRGL*_.*MU*_ and *PslRGA*_.*MU*_, highlighting the changes in DELLA and TVHYNP motifs generated by a site-directed mutagenesis approach. (**B**) Subcellular localization of full-length ORFs of PslRGL, PslRGA and their mutated derivatives fused to the GFP tag. All constructs were transiently transformed for the assay into *N*. *tabacum* protoplasts. *NLS*-mCherry was included in each transfection to indicate the location of the nucleus. GFP fluorescence is shown as green; the merged image is a digital merge of bright field and fluorescent images to illustrate the protein compartments. Bars = 10 μm. Interaction properties of PslRGL_.*MU*_ and PslRGA_.*MU*_ proteins with PslGID1s using Y2H (**C**) and BiFC (**D**) approaches. Corresponding native proteins were included as controls. All experiments were repeated a minimum of three independent times. Other details are as in Fig 2.

The previous results indicated that disrupted DELLA domain of PslRGA might potentially contribute for the lack of interaction with PslGID1-like proteins. To test this hypothesis, the active-interactor PslRGL and the non-active PslRGA proteins were subjected to a series of mutations. By comparing the amino acid residues of several DELLA sequences from different plant species, particularly between PslRGAa, PslRGAb and VvDLLA3, it appeared that Phe-35, Val-77 and Glu-86 residues are unique to PslRGAa (S4 Fig). Therefore, we mutated these three amino acids in PslRGAa into Leu, His and Try, respectively; to simulate active DELLAs. Similarly, the corresponding amino acids Leu-51, His-93 and Try-102 in PslRGL were changed into their analogs in PslRGAa. The mutated versions of both proteins were designated PslRGL_.*MU*_ and PslRGA_.*MU*_, respectively (Fig 4A). Assessing the localization of the two mutated versions revealed that both proteins remained targeting the nucleus compartment, indicating that the generated mutations did not affect their potential function as transcription regulators (Fig 4B). The consequences of these mutations were evaluated by assessing the changes in the dynamic of interaction property of the original ORFs (as a control) and ORFs carrying mutations, using both Y2H and BiFC approaches (Fig 4C and 4D). Relative to control ORFs, PslRGL_.*MU*_ protein lost the GA-dependent capacity to bind to any of the PslGID1 proteins. In contrast, PslRGA_.*MU*_ protein accomplished successful GA-dependent interaction, but only with PslGID1b protein. The previous results provided strong evidence that the changes within the DELLA domain of PslRGA is the cause of abolished interaction with PslGID1-like proteins.

### *PslDELLA* expression during fruit ontogeny

**Fig 5:**
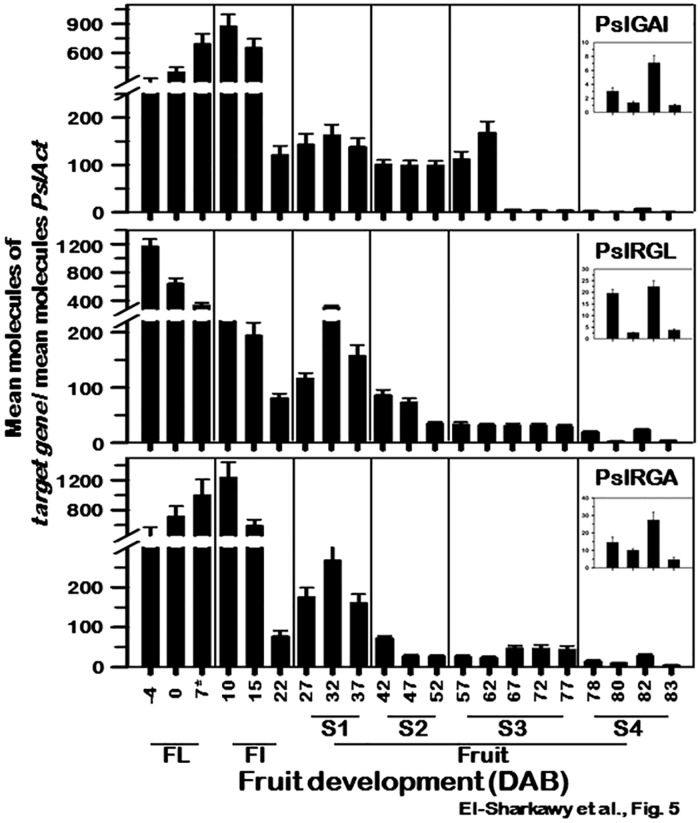
Steady-state transcript levels of *PslGAI*, *PslRGL* and *PslRGA* mRNAs assessed by qPCR during EG plum fruit development, including flowers (FL), fruit set (FI), and the 4 different stages of fruit development (S1-S4). Results represent data from three biological and three technical replicates. Standard curves were used to calculate the number of target gene molecules per sample. These were then normalized relative to *PslAct* expression. Error bars represent SD. The *y*-axis refers to the mean molecules of the target gene per reaction/mean molecules of *PslAct*. The *x*-axis in each figure represents the developmental stage as indicated by the number of days after bloom (DAB). The expression of the three genes during fruit ripening was over-exposed to visualize the changes in transcription levels.

Earlier studies reported that the expression of *DELLA* genes differ among various developmental stages. Whereas *AtRGA* and *AtGAI* are highly expressed in most tissues, *AtRGL1*, *AtRGL2*, and *AtRGL3* are mainly expressed in germinating seeds, young seedlings, and flowers [81]. Hence, the expression level of *PslDELLA* genes was assessed during various developmental stages to provide further credence about their role in regulating fruit growth. An initial screen of the five *PslDELLA* transcripts (*PslGAI*, *PslRGLa/b*, *PslRGAa/b*) indicated that all are expressed with no significant difference between the –a and –b gene pairing (data not shown). Consequently, the qPCR assays were performed on the –a gene variant of *PslRGL* and *PslRGA*. Although transcripts of *PslDELLA* were ubiquitously expressed, their accumulation profile appears to be organ- and developmental stage-dependent (Fig 5). This preliminary analysis indicated that these GA-negative signalling components might be transcriptionally regulated, as suggested for their orthologs in *Arabidopsis* [81].

All *PslDELLA* transcripts were abundantly expressed in flower buds (~ −4 DAB), but showed distinct accumulation pattern afterward. *PslRGL* transcripts gradually declined along with development, reaching low levels by the end of fruit initiation (~22 DAB). The signal of *PslRGL* detected in flower buds represents the highest abundance among the whole experiment. Conversely, *PslGAI* and *PslRGA* steadily increased along with flower development, peaking soon after fertilization, ~10 DAB. Subsequently, both transcripts behaved similarly to that of *PslRGL* mRNA by decreasing to their low levels at the end of fruit-set. GA is involved in diverse biological processes, particularly flowering and fruit initiation [21–22, 24]. It actively promotes flowering through regulating floral meristem identity genes [20] and floral integrator genes [23]. Similarly, GA is needed to organize the abundant cell division, expansion and embryo development during fruit-set phase [1–2]. The abundance of the three transcripts during flowering and fruit-set suggested a dominant task of *PslDELLA* in regulating GA-response during this stage. Recent evidence suggested that ethylene is involved in both the control of the ovule lifespan and the determination of the pistil/fruit fate. The proposed model suggests that ethylene may modulate the onset of ovule senescence and, consequently, the window of GA fruit-set responsiveness by altering GA-perception and -signalling. Though an actual mechanism remains unidentified, it was suggested that the ethylene produced in ovules would modulate the excessive GA-response by stabilizing the DELLAs via CTR1 [82–83]. Interestingly, a remarkable increase in the transcription of several ethylene-associated genes was eventually detected in plum during flower to fruit transition (i.e. in the same developmental stages used in the present study) [31, 67]. Genetic and biochemical analyses have shown that the five *AtDELLA* are actively involved in GA-signalling and they exhibit both overlapping and distinct roles in regulating GA-responsive growth [81, 84–86]. For instance, only RGA has been shown to prominently mediate GA effects on flower development, whereas GAI, RGA, RGL2 and RGL1 play the main role in the regulation of GA-dependent fruit initiation [44, 87–88]. The accumulation profile of the different *PslDELLA* suggested the contribution of the three transcripts in regulating floral meristem identity and flower bud initiation. However, only *PslGAI* and *PslRGA* are apparently more involved in mediating the GA-dependent events of fruit-set.

Stone fruits (*Prunus* spp.), including plum, exhibits a typical double sigmoid growth pattern during fruit development with four distinct stages; S1-S4 [13]. During S1 (27–37 DAB), the three transcripts increased to form a modest peak by ~32 DAB. Subsequently, *PslRGL* and *PslRGA* mRNAs gradually declined to reach relatively low levels by the end of S2 (~52 DAB); however that of *PslGAI* continue expressed at nearly constant moderate levels. Throughout fruit development, it is almost certain that the series of modifications that make the fruit proceed through the consequent developmental stages involve many different pathways, including the GA pathway. During S1, the GA is needed to organize the intense cell division and expansion [13]. In S2, there is hardly any increase in fruit size, as the fruit enter a period of growth dormancy. Therefore, the significant accumulation of *PslGAI* transcripts seemed to be associated with the lignification of the endocarp, the only developmental process occurring during this stage [9,13]. It was demonstrated that GA mediates lignin formation and deposition by polymerization of pre-formed monomers [89]. These results suggested that all *PslDELLA* should be active components of the GA-signal network that regulate fruit growth during immature S1 stage; however, only *PslGAI* is the dominant player in modulating the GA-responses during S2 phase. Comparing *PslDELLA* expression profile with the changes in GA contents from flowering until the end of S2-stage suggested that their accumulation is potentially associated with the growth signature events that are triggered in a GA-dependent manner, when GA-biosynthesis and -signalling actively occurred [9,13]. The abundance of *PslDELLA* in GA-rich tissues may be caused by rapid turnover of PslDELLA proteins or due to feedback regulation of *PslDELLA* transcription during active GA-signalling. Further, it was demonstrated that the GA-upregulated *OsSLR1* expression site is corresponding to the site of GA action, so its expression should be affected by GA levels [90].

During S3 maturation phase (57–77 DAB); the expression profile of the three transcripts remained slightly different. In early S3 stage (57–62 DAB), *PslGAI* signal was greatly detected and dramatically decreased to its basal levels afterward. However, those of *PslRGL* and *PslRGA* remained at low levels. Through S4, where most ripening-related metabolic changes occurred in an ethylene-dependent manner, all *PslDELLA* were scarcely detectable, signifying a minor contribution during mature growth phase. Interestingly, the decline in *PslDELLA* transcripts is associated with accelerated cell division and expansion events, resulting in visible enlargement in fruit size [13]. GA-mediated responses are under the tight regulation of growth-repressing DELLA proteins. According to the “relief of restraint” model, any activation of GA-signalling requires degradation of DELLA proteins [38, 44]. Therefore, the down-regulation of *PslDELLA* in mature fruiting tissues can enable fruit expansion and relief fruit growth, reaching their standard size. The effect of GA application in increasing fruit size and weight of several fruit species, including plum, strongly support this hypothesis [1, 13, 91].

Although, the levels of the three transcripts were hardly detected, a slight increase in their signal was observed at ~82 DAB. Remarkably, these minor increases coincided with the climacteric ethylene production peak [64]. Accordingly, it is tempting to speculate that *PslDELLA* are potentially regulated by ethylene during fruit ripening. To confirm this hypothesis, the expression of the three transcripts was assessed in EG fruit pre-treated with the ethylene stimulator propylene and the ethylene response inhibitor 1-MCP. The results provided further credence to the potential feedforward regulation of *PslDELLA* by ethylene in mature fruiting tissues (S5 Fig). As expected, propylene-treated fruit exhibited rapid and brief ripening profile in association with increased ethylene levels. In contrast, all fruit treated with 1-MCP were unable to ripen autonomously and their ethylene production remained low. Propylene treatment caused dramatic increase in all *PslDELLA* and this correlated well with the changes of ethylene production during fruit ripening (*R*^*2*^ = 0.96; *P*<0.01). Conversely, 1-MCP treatment abolished ethylene-induced *PslDELLA* expression. Previous studies suggested a cross-talk between GA and ethylene in the regulation of different aspects of plant development [82, 92]. However, the nature of interaction between the two hormones (positive or negative) is dependent on the developmental and environmental circumstances. Apparently, in mature fruit, ethylene alters GA-responses by directly or indirectly enhancing *DELLA* transcription and/or increasing DELLA proteins stability [8, 93].

### Overexpression of PslDELLA in WT *Arabidopsis*

**Fig 6:**
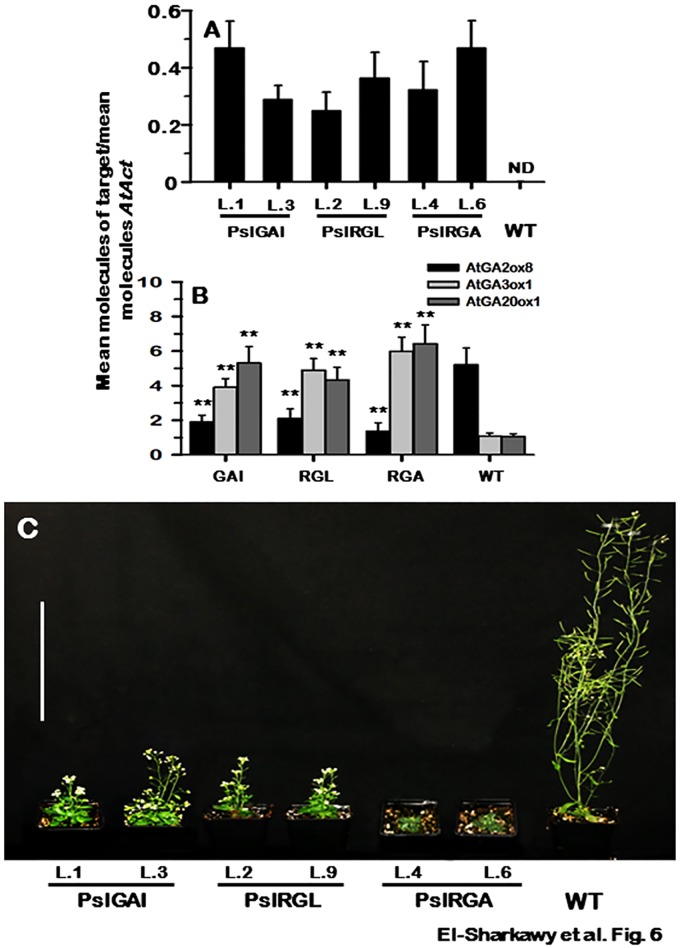
(**A**) *PslDELLA* transgene levels and (**B**) the accumulation of the GA-metabolism mRNAs in WT and the different transgenic events overexpressing *PslGAI* (L.1), *PslRGL* (L.9) and *PslRGA* (L.6) genes. Transcripts accumulation was determined using qPCR on three biological and three technical replicates. Standard curves were used to calculate the numbers of target gene molecules per sample, which were then normalized relative to *AtAct* expression. ND means non-detectable. (**C**) Aerial portions of 45-day-old WT and the different transgenic mutant plants grow under standard conditions; bars = 10 cm.

To examine PslDELLA-like protein function *in planta*, the three genes were introduced separately into WT *Arabidopsis* background (Col). A number of independent transgenic lines (15 to 26 lines / transformation) were obtained and confirmed by qPCR analysis. However, only two homozygous T3 representatives from each transformation were selected for further studies on the basis of differential transgene levels (Fig 6A). Transformed plants with empty vector were phenotypically indistinguishable from WT (data not shown). According to the model suggested by Achard and Genschik [78], DELLAs restrain plant growth, whereas GA promotes growth by targeting DELLAs for destruction. Therefore, increasing the amount of *DELLA*-repressors within the plant should lead to artefacts due to over-saturation in the system that typically affect the GA-signalling machinery, causing changes in plant phenotype consistent with aberrant DELLA protein accumulation [78, 94]. To better characterize the resultant phenotypes, the expression of some *Arabidopsis* genes involved in GA-metabolism was assessed. GA-homeostasis in a variety of plant species has been found to be tightly linked to the activities of enzymes involved in GA-biosynthesis and -catabolism [19, 95]. When GA levels and/or responsiveness are high, genes encoding enzymes for GA-biosynthesis (*AtGA20ox* and *AtGA3ox*) and enzymes for GA-inactivation (*AtGA2ox*) are subject to negative-feedback and positive-feedforward regulation, respectively [33]. Consistent with the GA-regulation model, the accumulation of *AtGA2ox8* strongly declined in *PslGAI*–, *PslRGL*–and *PslRGA*–plants by 64%, 60%, and 74%, respectively. While *AtGA20ox1* and *AtGA3ox1* steadily increased by ~5.1-, ~4.4-, and ~6.2-fold, and ~3.6-, ~4.2- and ~5.5-fold in *PslGAI*–, *PslRGL*–and *PslRGA*–plants, respectively (Fig 6B). The previous data suggested that the over-accumulation of *PslDELLA* in transgenic plants was able to alter the feedback and feedforward regulation of GA-metabolism pathway. We further characterized the molecular basis of the GA-signalling disturbance in transgenic plants by quantifying the levels of the five endogenous *AtDELLA* mRNAs. However, no significant differences between WT and transgenic plants in the levels of *AtDELLAs* were detected, confirming that the resulting phenotypes were due to the selective introduction of *PslDELLA* transgene (data not shown). Overexpression of *PslDELLAs* in WT *Arabidopsis* led to dramatic disturbances in general growth performance consistent with impaired GA-responses. All the plants overexpressing PslDELLA proteins exhibited a severe dwarf phenotype; however, the repressing activity of PslRGA was always much stronger than that of PslGAI and PslRGL proteins (Fig 6C).

### Developmental phenotypes of *PslDELLA* lines

Despite the advances in our understanding of the molecular basis of GA action, it remains unclear how these key phytohormones promote growth. Overexpressing *PslDELLA* in *Arabidopsis* visibly perturb overall plants growth behavior, including rooting capacity, plants architecture, and general vegetative and reproductive growth. Bioactive GA plays crucial roles in coordinating different plant growth aspects [5]. Thus, the interruption in GA-signalling pathway due to *PslDELLA*–overexpression can explain the distortion in different growth incidence of transgenic plants. Consequently, application of bioactive GA in such GA-deficient mutants has convenient implications in identifying the GA-dependent growth processes. Accordingly, the different *PslDELLA*–events were phenotypically characterized for some of well-known GA-dependent traits under standard growth conditions and in response to GA_3_ treatment.

**Fig 7:**
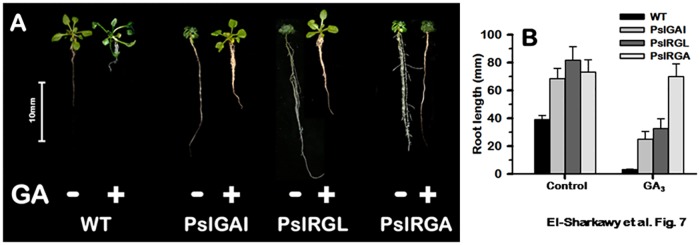
(**A**) Representative 15-day-old seedlings primary roots of WT, *PslGAI*, *PslRGL* and *PslRGA* genotypes. Plants were grown on MS medium in the presence or absence of GA_3_ (50 μM); bar = 10 mm. (**B**) Differential response of WT, *PslGAI*, *PslRGL* and *PslRGA* root growth to GA treatment. Root length measurements are the means (±SD) of 24 seedlings.

All *PslDELLA*–plants exhibited compact shoot growth associated with slender root formation and proliferation of lateral roots. The root length of *PslGAI*–, *PslRGL*–and *PslRGA*−plants were enhanced by ~ 0.7-, 1.1-, and 0.9-fold, respectively (Fig 7). By providing an exogenous supply of bioactive GA_3_ in the culture medium, we tested the GA-response of the independent *Arabidopsis* lines. Excluding *PslRGA*−plants, GA treatment caused a rapid stem elongation concomitant with a severe reduction in the formation of adventitious roots. By contrast, *PslRGA*−plants were unaffected by the treatment, producing compact shoots and elongated roots despite the GA_3_ incorporated in the medium.

The role of GA in plant development has been well characterized [5, 96]. Nevertheless, the way by how GA regulates plant development is still poorly understood [97–98]. The compact stem growth along with the accelerated root characteristics are a common behavior in GA-deficient mutants [47, 99–101]. Recent studies suggested that GA inhibited root growth by suppressing lateral root formation in a DELLA-dependent pathway [97, 101–103]. The conflict phenomenon of GA effects in shoot and root growth has been previously reported in several plants species [102–105]. The fact that *PslRGA* overexpression, as other *PslDELLA* promote lateral root formation, but selectively overcomes the inhibitory effect of GA on root formation support the idea that *PslRGA* encodes a functional DELLA-repressor, but insensitive to GA presence.

**Fig 8:**
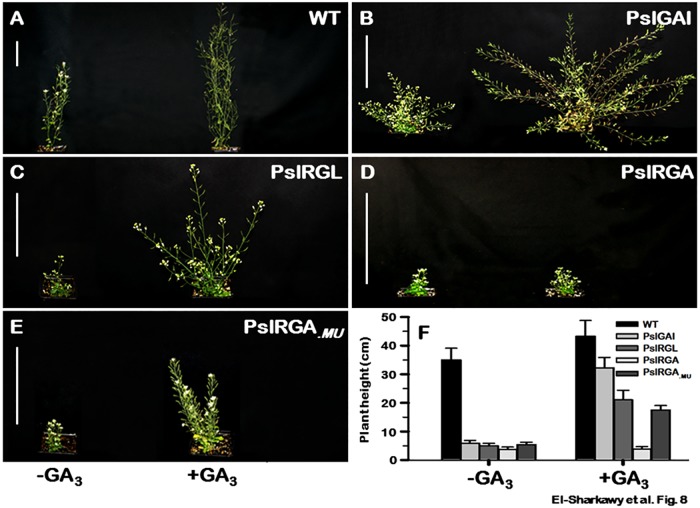
Representative 50-day-old aerial portions of WT (**A**), *PslGAI* (**B**), *PslRGL* (**C**), *PslRGA* (**D**) and *PslRGA*_.*MU*_ (**E**) plants grow under standard conditions with or without GA_3_ (100 μM) treatment; bars = 10 cm. (**F**) Differential response of WT, *PslGAI*−, *PslRGL*−, *PslGAI*− and *PslRGA*_.*MU*_−plant growth to GA treatment. Plant height measurements are the means (±SD) of 24 plants.

All transgenic *Arabidopsis* plants overexpressing the different *PslDELLA* genes showed compact growth due to substantial decline in the length of all stem growth-related characters, in which *PslRGA*–plants had the strongest dwarfing effect. Relative to WT, *PslGAI*–, *PslRGL*–and *PslRGA*–plants exhibited significant reduction in their overall heights by ~ 83%, 86% and 92%, respectively (Table 1, Fig 8). Moreover, *PslGAI*–and *PslRGL*–plants architecture was visibly different due to development of multiple branching architecture in association with numerous, but notably short internodes (Table 1, Fig 8B and 8C). Among the different transgenic events, *PslGAI*–plants displayed the highest branched structure followed by *PslRGL*–plants; however, such structure was not evident in *PslRGA*–plants. The altered branching pattern *PslDELLA*–plants is probably due to constitutive GA-response within the axillary bud meristem. Doust and his colleagues [106] have identified quantitative trait loci in foxtail millet for branching architecture, including genes encoding GA biosynthetic enzymes. In the LATERAL SUPPRESSOR (ls) mutant of tomato, in which axillary bud growth is repressed, the GA content of these buds is higher than in those of the wild type [107]. Apparently, PslDELLA-repressors alter plant structure directly or indirectly by triggering LS protein [37]. However, this pattern seems to be more associated with GA-sensitive DELLA-repressor.

**Table 1:** Vegetative and reproductive growth characteristics of WT *Arabidopsis* plants expressing plum *PslGAI*, *PslRGL*, *PslRGA*, *PslRGA*_.*MU*_ genes in the presence or absence of GA as shown in Figs 6 and 8.

		WT	PslGAI		PslRGL		PslRGA		PslRGA_.*MU*_	
			L.1	L.3	L.2	L.9	L.4	L.6	L.5	L.12
**Stem height (cm)** ^a^	**C**	**35(±1.4)**	5.2(±0.8)**	6.6(±0.9)**	5.3(±0.6)**	4.6(±1)**	3.1(±0.4)**	2.4(±0.8)**	4.8(±0.8)**	6(±0.8)**
	**GA**	**41.3(±2.1)**	25.4(±1.6)**	29.1(±2.2)**	15.8(±1.7)**	16.3(±2.5)**	2.9(±0.6)**	2.8(±1)**	14.4(±1.8)**	13.6(±1.5)**
**Length of internodes (mm)** ^a^	**C**	**27.6(±2.3)**	3.18(±0.5)**	4.09(±0.8)**	2.3(±0.5)**	2.12(±0.4)**	2.01(±0.4)**	1.09(±0.3)**	1.03(±0.18)**	1.05(±0.8)**
	**GA**	**38.2(±4.6)**	12.6(±1.3)**	13.8(±1.2)**	10.8(±1.6)**	8.32(±1.5)**	2.11(±0.3)**	1.18(±0.5)**	7.14(±1.1)**	7.66(±1.2)**
**No. of flowers** ^b^	**C**	**52.3(±10.5)**	23.1(±4.1)**	20.7(±3.7)**	5.5(±1.1)**	6.4(±1.6)**	4.4(±1.6)**	5.7 (±1)**	4.1(±1.6)**	3.6(±1)**
	**GA**	**78.2(±16.5)**	56.3(±9.4)**	58.5(±10.1)**	13.5(±2.1)**	17.4(±2.6)**	4.1(±1.2)**	5.5(±1.3)**	25.3(±2.4)**	27.9(±2.5)**
**Flowering time (d)** ^c^	**C**	**24.3(±1.2)**	42.3(±2.1)**	40.7(±1.5)**	45.7(±3.1)**	46.6(±3.2)**	56.3(±2.5)**	60.6(±3.5)**	61.2(±3.1)**	58(±2.1)**
	**GA**	**20.7(±1.7)**	33.1(±1.9)**	32.5(±2.1)**	34(±1.8)**	36.1(±2.6)**	58(±2.5)**	59.3(±3)**	42.7(±1.6)**	39.3(±1.5)**
**Pistil length (mm)** ^d^^,^^e^	**C**	**2.27(±0.11)**	0.98(±0.07)**	1.06(±0.09)**	0.82(±0.14)**	0.77(±0.15)**	0.69(±0.05)**	0.62(±0.16)**	0.63(±0.2)**	0.69(±0.09)**
	**GA**	**2.46(±0.05)**	1.99(±0.11)**	2.13(±0.07)**	1.71(±0.12)**	1.54(±0.07)**	0.67(±0.08)**	0.65(±0.11)**	1.58(±0.05)**	1.61(±0.1)**
**Stamen length (mm)** ^d^^,^^e^	**C**	**2.26(±0.14)**	0.61(±0.15)**	0.74(±0.08)**	0.51(±0.08)**	0.43(±0.07)**	0.32(±0.08)**	0.26(±0.06)**	0.34(±0.13)**	0.43(±0.06)**
	**GA**	**2.21(±0.1)**	1.85(±0.12)**	1.91(±0.07)**	1.62(±0.07)**	1.44(±0.09)**	0.37(±0.05)**	0.25(±0.07)**	1.44(±0.09)**	1.48(±0.17)**
**Silique length (mm)** ^a^^,^^e^	**C**	**13.52(±0.17)**	2.57(±0.12)**	3.09(±0.11)**	3.44(±0.11)**	2.75(±0.16)**	1.67(±0.1)**	1.48(±0.13)**	1.46(±0.06)**	1.49(±0.05)**
	**GA**	**10.88(±0.14)**	5.39(±0.15)**	6.43(±0.19)**	6.62(±0.17)**	5.58(±0.16)**	1.56(±0.13)**	1.45(±0.15)**	4.81(±0.09)**	4.96(±0.1)**
**Silique maturation time (d)**^a^^,^^f^	**C**	**12(±2)**	25.1(±1.5)**	24.8(±1.9)**	26.2(±1.5)**	28.9(±1.5)**	29.8(±1.6)**	30.6(±2.5)**	32(±2.8)**	31.2(±1.4)**
	**GA**	**15.5(±2.4)**	22.3(±1.6)**	21.5(±2.8)**	23.1(±3)**	24.4(±1.2)**	28.3(±2)**	29(±3)**	27.3(±1.5)**	26.3(±1.1)**
**No. of seeds/silique** ^a^^,^^e^	**C**	**38(±2)**	5(±1)**	6.7(±1.2)**	3.8(±1)**	3(±1.5)**	3(±1)**	2.3(±1.5)**	2.6(±1.2)**	3.7(±1.5)**
	**GA**	**29(±1)**	16.7(±1)**	18(±1.5)**	14.9(±1.1)**	13.8(±1.5)**	3.2(±1.3)**	2.5(±1.2)**	11(±1.7)**	10.3(±1)**

Plants were grown in LD photoperiod divided into two groups: control (C) and sprayed with 100μM of GA_3_ (GA). The measurements are the means (±SD) of approximately 24 plants. Statistical differences were assessed using Student’s *t*-test. Statistically significant differences from the WT with corresponding treatment are indicated by (*) and (**) for the probability levels (*P*<0.05) and (*P*<0.01), respectively. NS, non-significant (*P*>0.05).

^a^ The measurements were taken from adult plants have ~10% shattered siliques.

^b^ Number of flowers/inflorescence.

^c^ Flowering time was scored upon the emergence of first flower.

^d^ The measurements were taken from adult plants have ~10% shattered siliques.

^e^ The measurements are the average (±SE) of 12 flowers.

^f^ Silique maturation time was scored upon the appearance of the first shattered silique.

The transgenic plants differentially responded to the application of bioactive GA_3_ (Table 1; Fig 8F). GA treatment rescued to certain extent *PslGAI*–and *PslRGL*–plants’ height mainly due to extending internode length with no considerable changes in internode number. Relative to control untreated plants, no changes in the branch architecture were observed (Fig 8B, 8C and 8D). While, the compact phenotype of *PslGAI*–and *PslRGL*–mutants can be partially reversed by GA treatment, *PslRGA*–plants were not affected by GA presence (Table 1; Fig 8D and 8F) even after increasing the doses of GA to 500 and 1000 μM. With respect to the typical DELLA proteins with intact domains, putative DELLA-repressors holding disrupted DELLA domain are less responsive to GA-induced degradation, indicating that these proteins may function as constitutive suppressors of GA-signalling independent of GA action [49, 58–59, 90]. Thus, we hypothesize that the degenerated DELLA domain in *PslRGA* is the cause of GA-insensitive phenotype observed in corresponding plants. Apparently, such proteins operate to maintain a basal level of growth-restraint in specific tissues or at certain points of development despite the presence or absence of bioactive GA [108–109]. If this is correct, the generated version *PslRGA*_.*MU*_ with recovered DELLA domain should respond to GA treatment on the bases of its active interaction property detected in yeast system (Fig 4). Hence, transgenic *Arabidopsis* plants overexpressing *PslRGA*_.*MU*_ sequence were generated and its phenotypical growth characteristics in the presence or absence of GA_3_ were evaluated. Interestingly, *PslRGA*_.*MU*_–overexpression conferred a compact growth phenotype that is not obviously distinguishable from that occurred by the native *PslRGA* transgene (Table 1; Fig 8D and 8F). Contrary to *PslRGA*–plants, when *PslRGA*_.*MU*_–plants treated with GA they developed elongated internodes, resulting in partial recovery.

Plants can adopt a wide variety of environmental forms. The plasticity plays important roles in ecosystems, agriculture and landscape aesthetics. Under stressful circumstances, plants can rapidly respond to the environmental stimuli by rebuilding their system architecture to modify whole plant strategies, avoiding the environmental impact with maintaining productivity. The fundamental importance of these processes has prompted considerable research into how plants perceived the alert signal and how they governed the subsequent changes in growth behavior. Recent studies proposed that GA-signalling permits flexible and appropriate modulation of plant growth in response to changes in natural environments [63, 86, 93, 110]. Therefore, it is possible to speculate that GA is one of the key players that regulate the plant’s decision if exposed to unfavorable environmental conditions through upregulating DELLA-repressors, leading to impaired growth rate. If this is the case, it is obvious that the over-accumulation of *PslDELLA* in transgenic plants will turn-on the alert signal, resulting in not only reduced stem growth but also enhanced rooting system.

Gibberellins are involved in the developmental events leading to reproductive competence, as well as in floral determination and commitment [85, 93]. Under standard growth conditions, *PslDELLA*–overexpression caused substantial disorder in all phenotypical and phenological characteristics of reproductive growth. GA treatment was able to recover the different aspects of reproductive growth disruption in all mutants, excluding those of *PslRGA*–plants; however, the recovery remained visibly less than WT. Conversely, *PslRGA*–plants continued showing insensitive response to GA.

**Fig 9:**
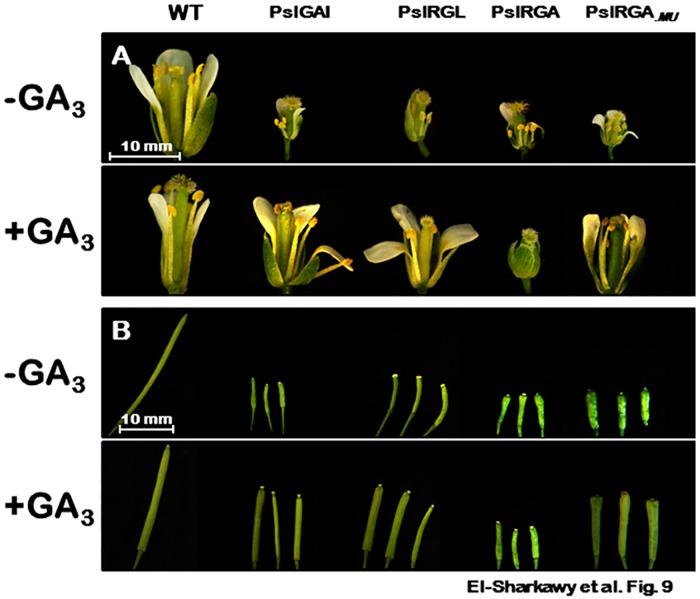
Close-up views of WT, *PslGAI*−, *PslRGL*−, *PslRGA*−, and *PslRGA*_.*MU*_−flowers (A) and siliques (B) from plants grow under standard conditions with or without GA_3_ (100 μM) treatment. Sepals and petals were removed to reveal the anthers and pistil; bars = 10 mm.

The number of flowers/inflorescence noticeably decreased in different transgenic events. In addition, the transition to flowering was considerably delayed in *PslGAI*–, *PslRGL*–, and *PslRGA*–plants by ~17, ~22, and ~35 days, respectively. Further, *PslDELLA*–plants displayed generally much smaller flower size and their filaments were usually shorter than their pistil (Table 1, Fig 9A). Such variation between the stamens and pistil can cause a major reduction in fertility, especially in self-pollinated species. Excluding *PslRGA*–flowers, GA application restored flowers number, flowering time and proper flower structure (Table 1).

Relative to WT, the time from flowering to silique maturation was significantly delayed by ~ 13, 16 and 19 days in *PslGAI*–, *PslRGL*–, and the two *PslRGA*–related plants, respectively (Table 1). GA treatment slightly delayed silique maturity in WT plants. This contradictory response is probably due to reach over-dose levels of the hormone, suggesting the importance of optimal GA levels to ensure proper growth and development. By contrast, the treatment considerably reduced siliques shattering duration, but substantially remained longer than WT. Furthermore, both silique length and seed number were drastically reduced in *PslDELLA*–plants (Table 1, Fig 9B). Silique lengths of *PslGAI*–, *PslRGL*–, and the two *PslRGA*–related plants were reduced by ~ 79%, 77% and 89%, respectively. Although all mutants exhibited significant reduction in seed number potentially due to compromised flower structure, the seeds were completely developed, but exhibited delayed germination estimated by 2 days later than WT in all *PslDELLA*–mutants. Analysis of different growth aspects in response to GA application highlighted the *PslRGA*_.*MU*_–plants as the strongest mutant resisting the stimulatory effect of GA in re-establishing typical growth, suggesting the existence of other obstacles within the sequence that still impairs PslRGA_.*MU*_–AtGID1 interactions.

### GA-insensitivity due to PslRGA stability

Ultimate GA-response is the result of antagonistic reaction between GA activation and suppression mechanisms [111]. Therefore, any disturbance in these machineries can modify the response of plants to active GA. The GA-insensitivity observed in transgenic *PslRGA*–plants along with the ability of GA treatment to rescue *PslRGA*_.*MU*_–plants suggested that PslRGA is a highly stable DELLA protein. To confirm this hypothesis, we determined the kinetics change in detectable level of nuclear PslDELLA−GFP fluorescence in transgenic *Arabidopsis* roots independently expressing the different PslDELLA−GFP proteins after 60, 120 and 240 minutes of GA_3_ treatment. Using this approach allowed us to monitor any alteration in the dynamic of PslDELLA−GFP proteins degradation in response to exogenously applied GA, which is informative because PslDELLA−GFP proteins are functionally active in respective transgenic plants.

**Fig 10:**
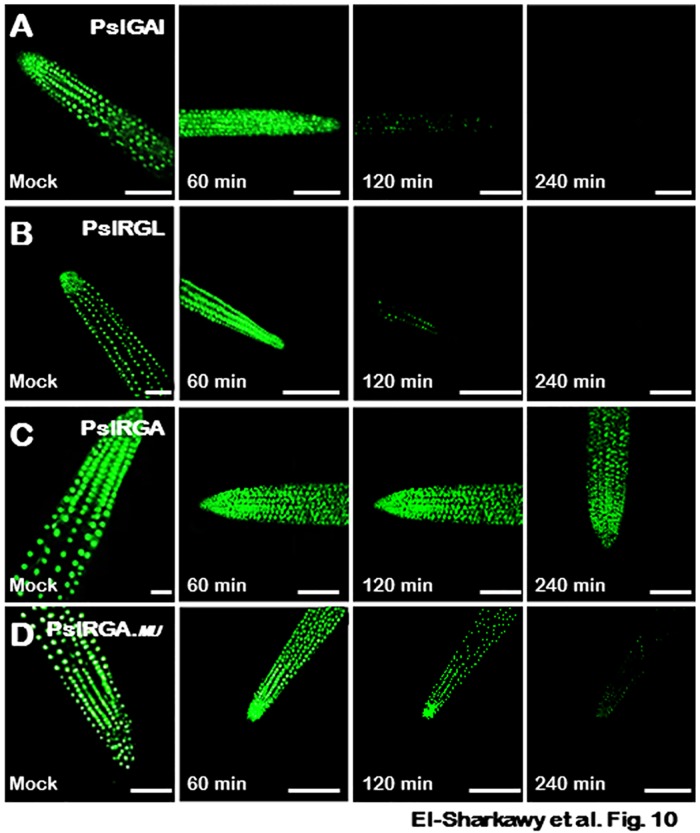
PslRGA is not subjected to GA-induced degradation. GFP fluorescence of primary 5-day-old *Arabidopsis* seedling roots, expressing (**A**) *PslGAI*−GFP, (**B**) *PslRGL*−GFP, (**C**) *PslRGA*−GFP, and (**D**) *PslRGA*_.*MU*_−GFP. Fluorescence were monitored with confocal laser scanning microscopy after treatment with water (mock) or GA_3_ (100 μM) for 60, 120 and 240 minutes.

Fluorescence intensity of *PslGAI*–and *PslRGL*−GFP chimeric proteins in root cell nuclei decreased substantially within 120 minutes of GA treatment and disappeared after 240 minutes, indicative of complete degradation of both proteins (Fig 10A and 10B), as demonstrated previously for several GA-sensitive DELLA proteins [63, 112–114]. Conversely, this dynamic GA-induced degradation of the two PslDELLA proteins was not seen in transgenic roots carrying PslRGA. *PslRGA*−GFP fluorescence showed full resistance to the destabilizing impact of GA and did not display any changes in fluorescence intensity 240 minutes after the onset of GA treatment (Fig 10C).

The stability of PslRGA protein was further confirmed by assessing the response of modified *PslRGA*_.*MU*_−GFP expressed in *Arabidopsis* root tips to GA treatment. Although *PslRGA*_.*MU*_−GFP showed slower degradation rate in response to GA presence comparing with PslGAI and PslRGL proteins, the generated protein was readily degradable and almost disappeared 240 minutes post GA treatment (Fig 10D). One possible explanation for the relative persist signal of *PslRGA*_.*MU*_−GFP for longer period post GA treatment would be attributable to the existence of other substitutions within the sequence that impair PslRGA_.*MU*_–AtGID interactions.

**Fig 11:**
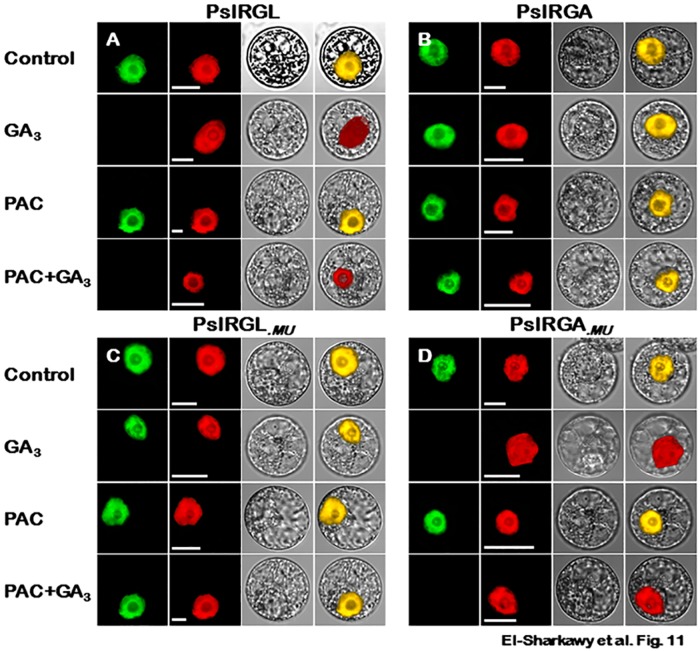
Modifications in *PslRGL* and *PslRGA* DELLA domain alter their GA-dependent responses. Confocal microscopic images of GFP fluorescence in tobacco *BY*-2 cells transiently expressed *PslRGL*−GFP (**A**), *PslRGA*−GFP (**B**), *PslRGL*_.*MU*_−GFP (**C**) and *PslRGA*_.*MU*_−GFP (**D**) chimeric proteins. *BY*-2 cells were treated with GA_3_ (100 μM), paclobutrazol (10 μM) and a joint treatment of PAC and GA. Non-treated cells were used as control. *NLS*-mCherry was included in each transfection to indicate the location of the nucleus. GFP fluorescence is shown as green; the merged image is a digital merge of bright field and fluorescent images to illustrate the protein compartments. All experiments were repeated a minimum of three independent times; bars = 10 μm.

In subsequent experiments, we transiently expressed the native ORFs *PslRGL*−GFB and *PslRGA*−GFP as well as their modified derivatives (*PslRGL*_.*MU*_−GFP and *PslRGA*_.*MU*_−GFP) in tobacco protoplasts treated with GA, paclobutrazol (PAC), and a joint treatment of PAC followed by GA to avoid the involvement of endogenous GA effect. Consistent with the previous results, the application of GA to protoplasts carrying the *PslRGL*−GFP and *PslRGA*_.*MU*_−GFP derivatives induced the disappearance of GFP florescence signal (Fig 11A and 11D). However, the intensity of nuclear signal in protoplasts transfected with *PslRGA*−GFP and more interestingly that of *PslRGL*_.*MU*_−GFP holding degenerated DELLA domain was not affected by GA (Fig 11B and 11C). Because PAC inhibits GA-biosynthesis, we sought to determine whether PAC treatment would have a different effect on PslDELLA protein degradation. PAC treatment enhanced the protein stabilization, as determined by the strong GFP signal detected with all tested chimeric proteins (Fig 11). We then examined the response of different proteins to a combined treatment of PAC+GA to confirm that the rapid loss of GFP fluorescence is GA-dependent. The responses of different proteins to the combined treatment were similar to their response to protoplasts treated with GA only (Fig 11). Thus, GA activity seemed to cause the reduced level of the PslRGL and PslRGA_.*MU*_ proteins. These results further support the critical contribution of conserved DELLA domain in mediating the GA-dependent DELLA degradation. The stability of DELLA proteins was demonstrated previously for plant mutants exhibiting naturally occurred and/or intentionally induced mutations (point mutation, deletion or truncation) within the critically important DELLA domain [58, 90, 94, 103, 115–118]. The absence of the conserved DELLA domain abolished the interaction of corresponding proteins with GID1, affecting their subsequent degradation via the ubiquitin/26S proteasome pathway [53–54].

The consistency of PslDELLA responses to GA using different approaches suggested that plum fruit development is actively regulated by three types of DELLA transcription factors in which two of them encode GA-sensitive proteins (PslGAI and PslRGL); however, the third one is GA-insensitive (PslRGA). This raises the question—why plum trees comprise a GA-insensitive DELLA protein within its genome? The destabilization of PslGAI and PslRGL likely released the growth-restraining effects of the two proteins in the fruiting tissues. It could be speculated that because DELLA-less forms of proteins, as PslRGA, are more resistant to GA-dependent degradation, they will have strong effect in controlling GA-signalling that coordinate fruit growth. Thus, such proteins can be present in plants and function as a backup system in place, avoiding the unnecessary excessive accumulation of GA-signalling. Our observation that *PslRGA*−overexpression inhibits the expansion of reproductive growth, overcoming the destabilization effect of GA application supports this hypothesis.

## Supporting Information

S1 Fig Evolutionary relationships of DELLA proteins. The evolutionary distances were computed using the Poisson correction method. The analysis involved 31 amino acid sequences from different plant species that belong to monocots and dicots, including *P*. *salicina* (Psl), *P*. *persica* (Pp), *P*. *mume* (Pm), *M*. *domestica* (Md), *F*. *vesca* (Fv), *V*. *vinifera* (Vv), *S*. *lycopersicum* (Sl), *A*. *thaliana* (At), *P*. *trichocarpa* (Pt), *O*. *sativa* (Os) and *Z*. *mays* (Zm). Bootstrap confidence values from 1000 replicates are indicated above branches.
(DOCX)

S2 Fig Subcellular localization of *PslDELLA* sequences fused to the GFP tag. All constructs were transiently transformed for the assay into *N*. *tabacum* protoplasts. *NLS*-mCherry was included in each transfection to indicate the location of the nucleus. GFP fluorescence is shown as green; the merged image is a digital merge of bright field and fluorescent images to illustrate the protein compartments. All experiments were repeated a minimum of three independent times; bars = 10 μm.
(DOCX)

S3 Fig The *3-D* modelling structure of PslGAI, PslRGL, PslRGA, and the *Arabidopsis* AtGAI proteins. The hydrophobic, polar, positively-, and negatively-charged residues are indicated in white, green, blue and red colors, respectively.
(DOCX)

S4 Fig Alignment of amino acid sequences of the GA-insensitive PslRGAa, PslRGAb (uncharacterized) and their closest GA-sensitive paralog in grape *VvDELLA3*. Amino acid residues in red represent the three amino acids mutated for functional analysis. Other details as in Fig 1.
(DOCX)

S5 Fig Ethylene production and steady-state *PslDELLA* levels during four different ripening stages [non-climacteric (NC), pre-climacteric (PrC), climacteric (C) and post-climacteric (PoC)] in control EG fruit and fruit pre-treated with propylene (1000 μl l^–1^) and the ethylene-inhibitor 1-MCP (1 μl l^–1^). Mature EG fruit (76 DAB) were harvested before autocatalytic ethylene production had risen and subjected to various treatments. Other details as in Fig 5.
(DOCX)

S1 Table The oligonucleotide primers. (DOCX)

S2 Table Amino acid sequence comparison between the predicted full-length plum and *Arabidopsis DELLA* gene family. (DOCX)

## Abbreviations

BiFC: Bimolecular fluorescence complementation
EG: Early Golden
PAC: paclobutrazol
S1-S4: Stage 1–4
WT: wild-type
Y2H: Yeast two-hybrid
