# Supplementary material for: Plum Fruit Development Occurs via Gibberellin–Sensitive and –Insensitive DELLA Repressors
Source: PLoS One. 2017 Jan 11;12(1):e0169440. doi: 10.1371/journal.pone.0169440 (PMC5226729; doi:10.1371/journal.pone.0169440)

**S1 Fig.** Evolutionary relationships of DELLA proteins. The evolutionary distances were computed using the Poisson correction method. The analysis involved 31 amino acid sequences from different plant species that belong to monocots and dicots, including *P. salicina* (Psl), *P. persica* (Pp), *P. mume* (Pm), *M. domestica* (Md), *F. vesca* (Fv), *V. vinifera* (Vv), *S. lycopersicum* (Sl), *A. thaliana* (At), *P. trichocarpa* (Pt), *O. sativa* (Os) and *Z. mays* (Zm). Bootstrap confidence values from 1000 replicates are indicated above branches.


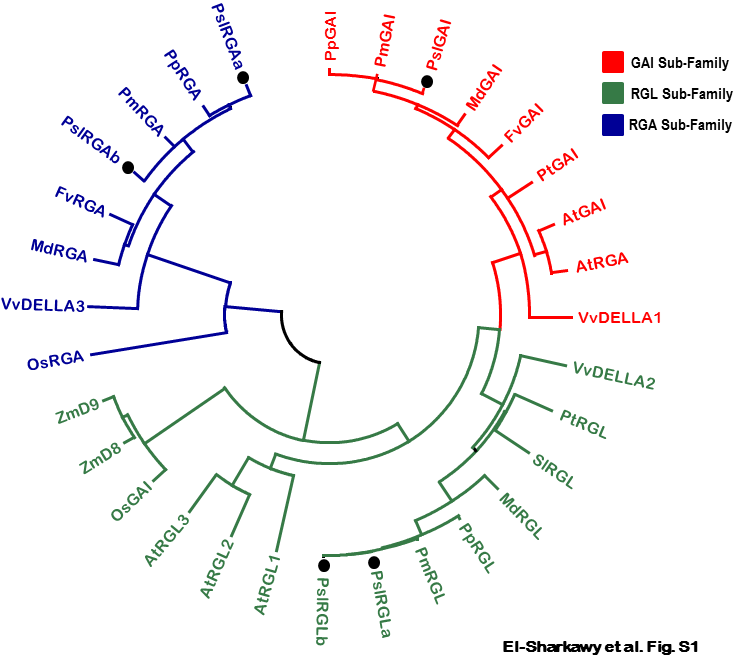

Supplement: S1 Fig — The evolutionary distances were computed using the Poisson correction method. The analysis involved 31 amino acid sequences from different plant species that belong to monocots and dicots, including P. salicina (Psl), P. persica (Pp), P. mume (Pm), M. domestica (Md), F. vesca (Fv), V. vinifera (Vv), S. lycopersicum (Sl), A. thaliana (At), P. trichocarpa (Pt), O. sativa (Os) and Z. mays (Zm). Bootstrap confidence values from 1000 replicates are indicated above branches. (DOCX) [file pone.0169440.s001.docx]
