# Supplementary material for: Plum Fruit Development Occurs via Gibberellin–Sensitive and –Insensitive DELLA Repressors
Source: PLoS One. 2017 Jan 11;12(1):e0169440. doi: 10.1371/journal.pone.0169440 (PMC5226729; doi:10.1371/journal.pone.0169440)

**S2 Fig.** Subcellular localization of *PslDELLA* sequences fused to the GFP tag. All constructs were transiently transformed for the assay into *N. tabacum* protoplasts. *NLS*-mCherry was included in each transfection to indicate the location of the nucleus. GFP fluorescence is shown as green; the merged image is a digital merge of bright field and fluorescent images to illustrate the protein compartments. All experiments were repeated a minimum of three independent times; bars=10 μm.


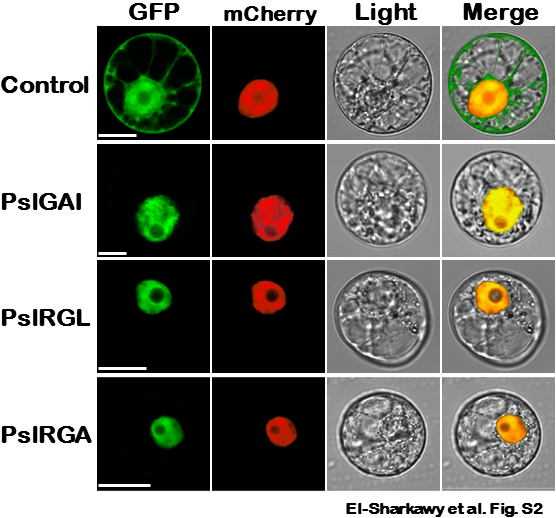

Supplement: S2 Fig — All constructs were transiently transformed for the assay into N. tabacum protoplasts. NLS-mCherry was included in each transfection to indicate the location of the nucleus. GFP fluorescence is shown as green; the merged image is a digital merge of bright field and fluorescent images to illustrate the protein compartments. All experiments were repeated a minimum of three independent times; bars = 10 μm. (DOCX) [file pone.0169440.s002.docx]
