# Supplementary material for: Plum Fruit Development Occurs via Gibberellin–Sensitive and –Insensitive DELLA Repressors
Source: PLoS One. 2017 Jan 11;12(1):e0169440. doi: 10.1371/journal.pone.0169440 (PMC5226729; doi:10.1371/journal.pone.0169440)

**S3 Fig.** The *3-D* modelling structure of PslGAI, PslRGL, PslRGA, and the *Arabidopsis* AtGAI proteins. The hydrophobic, polar, positively-, and negatively-charged residues are indicated in white, green, blue and red colors, respectively.


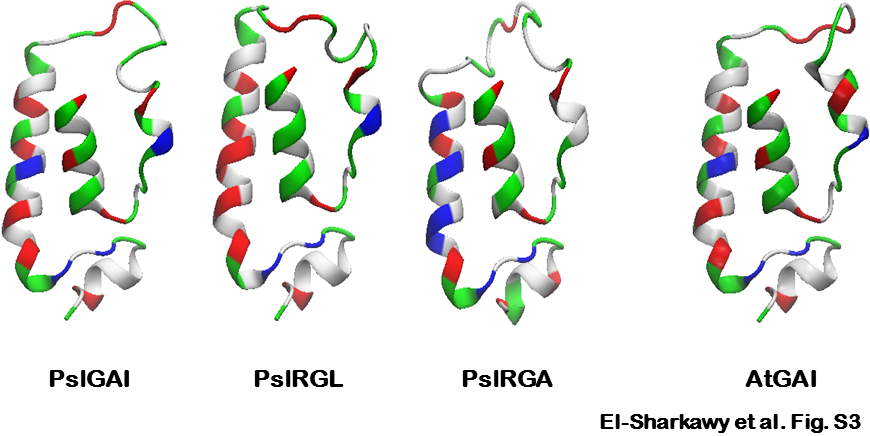

Supplement: S3 Fig — The hydrophobic, polar, positively-, and negatively-charged residues are indicated in white, green, blue and red colors, respectively. (DOCX) [file pone.0169440.s003.docx]
