# Supplementary material for: Plum Fruit Development Occurs via Gibberellin–Sensitive and –Insensitive DELLA Repressors
Source: PLoS One. 2017 Jan 11;12(1):e0169440. doi: 10.1371/journal.pone.0169440 (PMC5226729; doi:10.1371/journal.pone.0169440)

**S4 Fig.** Alignment of amino acid sequences of the GA-insensitive PslRGAa, PslRGAb (uncharacterized) and their closest GA-sensitive paralog in grape *VvDELLA3*. Amino acid residues in red represent the three amino acids mutated for functional analysis. Other details as in Fig.1.


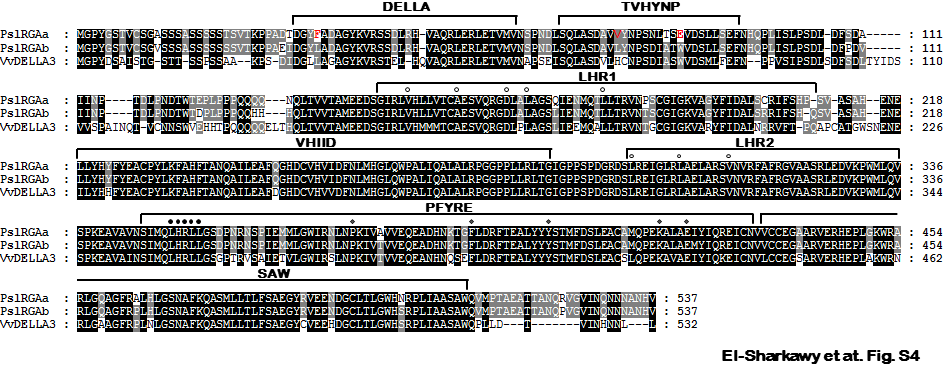

Supplement: S4 Fig — Amino acid residues in red represent the three amino acids mutated for functional analysis. Other details as in Fig 1. (DOCX) [file pone.0169440.s004.docx]
