# Supplementary material for: Plum Fruit Development Occurs via Gibberellin–Sensitive and –Insensitive DELLA Repressors
Source: PLoS One. 2017 Jan 11;12(1):e0169440. doi: 10.1371/journal.pone.0169440 (PMC5226729; doi:10.1371/journal.pone.0169440)

**S5 Fig.** Ethylene production and steady-state *PslDELLA* levels during four different ripening stages [non-climacteric (NC), pre-climacteric (PrC), climacteric (C) and post-climacteric (PoC)] in control EG fruit and fruit pre-treated with propylene (1000 μl l^–1^) and the ethylene-inhibitor 1-MCP (1 μl l^–1^). Mature EG fruit (76 DAB) were harvested before autocatalytic ethylene production had risen and subjected to various treatments. Other details as in figure 5.


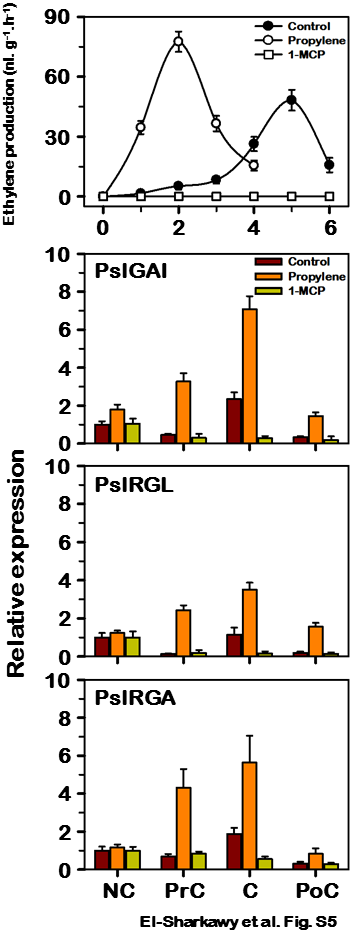

Supplement: S5 Fig — Mature EG fruit (76 DAB) were harvested before autocatalytic ethylene production had risen and subjected to various treatments. Other details as in Fig 5. (DOCX) [file pone.0169440.s005.docx]
