# Supplementary material for: Plum Fruit Development Occurs via Gibberellin–Sensitive and –Insensitive DELLA Repressors
Source: PLoS One. 2017 Jan 11;12(1):e0169440. doi: 10.1371/journal.pone.0169440 (PMC5226729; doi:10.1371/journal.pone.0169440)
Supplement: S1 Table — (DOCX) [file pone.0169440.s006.docx]

**S1 Table.** The oligonucleotide primers.

|  | |
| --- | --- |
| Name | Oligonucleotide sequence |
| *P. salicina* | |
| **1-DEL(F)** | **5’-**AAATTCGCTCACTTCACCGCCAATCA**-3’** |
| **2-DEL(R)** | **5’-**CATACTCGCCTGCTTGAACGCGTTCGA**-3’** |
| **3-qGAI(F)** | **5’**-GGAAGAAGATGCCCAACGAA-**3’** |
| **4-qGAI(R)** | **5’**-CCAACACGGCCAGAAGCT-**3’** |
| **5-qRGL(F)** | **5’-**TGGTTCCAAGGGCGAATA-**3’** |
| **6-qRGL(R)** | **5’-**CGCAGCCATCTTGGTCTT-**3’** |
| **7-qRGA(F)** | **5’**-ACTTCCCTGACGTCATCATCAA-**3’** |
| **8-qRGA(R)** | **5’**-GAGGTTCTGTCCAGGTGTCGTT-**3’** |
| **9-qAct(F)** | **5’**-CTGGACCTTGCTGGTCGT-**3’** |
| **10-qAct(R)** | **5’**-ATTTCCCGCTCAGCAGTG-**3’** |
| *A. thaliana* | |
| **11-qGA2ox8(F)** | **5’**-GATGAACCGATATCCACCTTGTC-**3’** |
| **12-qGA2ox8(R)** | **5’**-CTGTCCGTGTGTGGCATTAATC-**3’** |
| **13-qGA3ox1(F)** | **5’**-CACTACCCGGTTTGTCCTGAA-**3’** |
| **14-qGA3ox1(R)** | **5’**-GTGGAGTCGGTATGAGCTGCTA-**3’** |
| **15-qGA20ox1(F)** | **5’**-CGGACGCTTCTCCACCAA-**3’** |
| **16-qGA20ox1(R)** | **5’**-CGTCGCAAAACCGGAAAG-**3’** |
| **17-qAtAct(F)** | **5’**-TCCGTTGTCCTGAGGTTCTGT-**3’** |
| **18-qAtAct(R)** | **5’**-ATGGATTCCAGGAGCTTCCA-**3’** |
