# Supplementary material for: Plum Fruit Development Occurs via Gibberellin–Sensitive and –Insensitive DELLA Repressors
Source: PLoS One. 2017 Jan 11;12(1):e0169440. doi: 10.1371/journal.pone.0169440 (PMC5226729; doi:10.1371/journal.pone.0169440)
Supplement: S2 Table — (DOCX) [file pone.0169440.s007.docx]

**S2 Table.** Amino acid sequence comparison between the predicted full-length plum and *Arabidopsis* *DELLA* gene family.

|  | | | | | | |
| --- | --- | --- | --- | --- | --- | --- |
|  | **Amino acid sequence similarity (%)** | | | | | |
|  | **Protein size**  (No. of aa) | **PslGAI** | **PslRGLa** | **PslRGLb** | **PslRGAa** | **PslRGAb** |
| *P. salicina* |  |  |  |  |  |  |
| **PslGAI** | 633 | - | 70 | 69 | 52 | 52 |
| **PslRGLa** | 593 |  | - | 99 | 55 | 55 |
| **PslRGLb** | 596 |  |  | - | 54 | 55 |
| **PslRGAa** | 537 |  |  |  | - | 96 |
| **PslRGAb** |  |  |  |  |  | - |
|  |  |  |  |  |  |  |
| *A. thaliana* |  |  |  |  |  |  |
| **AtGAI** | 532 | 66 | 67 | 66 | 56 | 57 |
| **AtRGA** | 588 | 68 | 68 | 67 | 53 | 54 |
| **AtRGL1** | 511 | 61 | 70 | 69 | 55 | 56 |
| **AtRGL2** | 547 | 62 | 69 | 69 | 57 | 57 |
| **AtRGL3** | 523 | 59 | 67 | 66 | 56 | 58 |
